# Intervention Effectiveness of Health Behaviors During COVID‐19: A Systematic Review and a Network Meta‐Analysis

**DOI:** 10.1002/pchj.70054

**Published:** 2025-09-29

**Authors:** Ruihua Zhou, Kan Shi, Shuqi Li, Wei Zhou

**Affiliations:** ^1^ School of Education Shanghai Normal University Shanghai China; ^2^ Academy of Wenzhou Model Development, Educational School Wenzhou University Wenzhou China

**Keywords:** COVID‐19, health intervention, health‐promoting behavior, network meta‐analysis, preventive health behavior

## Abstract

In the context of a global public health crisis, such as COVID‐19, developing interventions to improve population health behaviors has emerged as a pivotal element of health management strategies. The efficacy of various interventions implemented during this period has varied, and the impact of different variables on these intervention outcomes remains to be fully elucidated. This study screened 57 papers (*n* = 47,264) by searching electronic databases and revealed the optimal intervention through pairwise meta‐analysis and network meta‐analysis, as well as the changes in intervention effectiveness under different conditions. Our research findings indicate that interventions for preventive health behaviors and health‐promoting behaviors have significant effects. For preventive health behaviors, the intervention method of health education and low‐risk information framework under information intervention was the optimal intervention. For health‐promoting behaviors, the exercise intervention and the prosocial information framework with information intervention were the optimal interventions. Accordingly, future research should focus on the in‐depth exploration of specific interventions to establish and improve the effectiveness of interventions.

## 1 Introduction

Over the four years following the COVID‐19 outbreak, there has been a significant transformation in people's health‐related awareness and behavioral patterns. During the epidemic's early stages, various countries and regions implemented policies and healthcare guidelines to mitigate its spread. These measures included social distancing, masking, and vaccination (World Health Organization 2020). Research shows that these non‐pharmacological interventions were significantly effective in preventing COVID‐19 (Chu et al. 2020; Spinelli et al. 2021). As COVID‐19 has evolved, people's preventive and health behaviors have also significantly improved, reflecting an enhanced societal capacity to adapt and respond to public health crises.

In existing epidemiological studies, intervention programs drawing on psychological, economic, and sociological theories have been implemented. This includes promoting health behaviors through a variety of informational frameworks (Rothman and Salovey 1997) and enhancing health and preventive behaviors using psychological approaches (Harris and Epton 2009). However, in the context of COVID‐19, current research has yet to explore the most effective interventions for health behaviors.

To systematically assess the efficacy of diverse interventions and identify which are most effective, we will employ network meta‐analysis (NMA). The advantage of NMA over traditional pairwise meta‐analysis lies in its ability to include and compare multiple interventions for the same outcome, systematically and comprehensively assessing the relative merits of each intervention through visual evidence (Mills et al. 2013). This approach aims to reduce irrational behaviors driven by virus fears, provide more scientifically grounded guidance on health and prevention behaviors, offer protection against potential new and unknown epidemic threats, and enhance overall human health awareness.

### 1.1 Health Behavior

The concept of health behavior is frequently discussed across various domains. Kirscht (1983) conceptualizes preventive health behavior as an individual's natural response to safeguard their health or mitigate potential risks and adverse effects in their surroundings. These behaviors serve as primary prevention measures until more effective treatments are developed. During COVID‐19, the main preventive health behaviors include wearing masks, washing hands, getting vaccinated, and keeping social distance (Callaghan et al. 2021). In contrast, health‐promoting behaviors are actions aimed at enhancing happiness, personal fulfillment, and longevity, ultimately improving the quality of life (Bhandari and Kim 2016). Health‐promoting behaviors include physical activity and healthy dietary behaviors, which help enhance immunity against viral infections. These behaviors have proven to be consistently effective, even during COVID‐19 (Tavakol et al. 2021). Pan (2020) noted that in addition to regular hand washing, appropriate exercise to enhance bodily immunity can serve as one of the long‐term health management strategies in response to COVID‐19. Considering the unpredictable potential of future virus outbreaks, it is crucial to explore physical activity, exercise, and other health‐promoting behaviors as innovative, long‐term strategies for combating the virus and enhancing public health (Souza Filho and Tritany 2020). Based on the discussion above, this paper identifies preventive health behaviors and health‐promoting behaviors as the primary health behaviors during COVID‐19.

### 1.2 The Present Study of Health Behavior Intervention

To date, numerous meta‐analyses and systematic reviews have examined the effectiveness of health behavior interventions across various contexts (Conroy and Hagger 2018; Ntoumanis et al. 2021). Early comprehensive reviews, such as that by Kirscht (1983), have demonstrated that interventions targeting general preventive health behaviors are relatively effective. However, with the development of society, technology, and the Internet, the effectiveness of various intervention measures is gradually changing. Additionally, previous meta‐analyses have predominantly concentrated on health behaviors in routine settings or within specific populations (Krebs et al. 2010; Plotnikoff et al. 2015), with limited emphasis on intervention effectiveness during infectious disease outbreaks. Recent methodological advances have highlighted network meta‐analysis (NMA) as a promising approach in the domain of health promotion. Nevertheless, this approach has rarely been applied in epidemic contexts.

COVID‐19 provides a unique and unprecedented context for exploring health behavior interventions. Unlike routine or general preventive health situations, pandemics generate distinct psychological and behavioral responses, amplifying existing challenges and revealing novel intervention needs (Brooks et al. 2020). During COVID‐19, previous meta‐analyses have explored the effect sizes of individual preventive behaviors and their related factors, but they have lacked the discussion of intervention measures (Li et al. 2022). Additionally, although previous studies have confirmed the efficacy of health behavior interventions across various contexts, their applicability within the unique context of COVID‐19 needs further investigation. Therefore, this study uses network meta‐analysis (NMA) to compare the effectiveness of different health behavior interventions during COVID‐19. Theoretically, it examines whether existing health behavior theories remain applicable during public health emergencies. Practically, this study aims to guide individuals in protecting their health during pandemics and to provide insights for responding effectively to future health crises.

## 2 Methods

### 2.1 Searching Strategy

This meta‐analysis was conducted in January 2023 with searches of the following databases, including PubMed, Web of Science, and ScienceDirect, supplemented by searches using PsycINFO, PsycARTICLES, and Google Scholar, through August 2023. The keywords in the title or abstract mainly contained the following: COVID‐19, health behavior, preventive behavior, preventive health behaviors, disinfecting, social distancing, physical activity, hand washing, face masks, eye protection, and wearing masks. The specific search strategy will be visible in Data S1. This study adhered to the Preferred Reporting Items for Systematic Reviews and Meta‐Analyses (PRISMA) guidelines.

### 2.2 Inclusion and Exclusion Criteria

We incorporate the following inclusion criteria for literature selection to ensure alignment with the requirements of this paper and achieve the intended purpose:

(1) Population: Studies focusing on the general public during COVID‐19, including individuals with physical or mental health conditions; literature published between January 2020 and April 2023 was retrieved. (2) Intervention: Health‐promoting or preventive interventions targeting COVID‐19–related behaviors. (3) Comparator: Only studies with a control group (e.g., no‐treatment, usual care, or another type of comparator) are included; studies without a control group are excluded. (4) Outcomes: The primary outcome is COVID‐19–specific health behavior, with available data on means, standard deviations, and sample sizes. (5) Study Design: Randomized controlled trials (RCTs) or quasi‐experimental studies are accepted; systematic reviews and meta‐analyses are excluded.

For studies suspected of using the same sample, the articles were further scrutinized to ensure that the results were not from the same sample. For those studies with similar or identical dependent variable indicators in the same study, the practice of using mean effect size was adopted in the NMA (Borenstein et al. 2021). For articles with missing data, we attempted to contact the corresponding authors, and articles that did not receive corresponding data responses were not included. The meta‐analysis studies included are in the Data S3.

### 2.3 Data Extraction and Quality Assessment

Before formal data extraction, three investigators (ZR, LS, and ZW) underwent a one‐month training program in data coding. Subsequently, they independently conducted literature screening using EndNote software and independently extracted and coded data using a standardized template (in Data S4). Any discrepancies encountered during this process were discussed with SK until consensus was reached. The final coding consistency was 100%. The extracted data included: study authors, year of publication, mean and standard deviation of baseline and endpoint for the experimental or treatment group versus the control group, country/region, dependent variable, study design, mean age and gender ratio.

Quality assessment was independently conducted by ZR and LS, with any discrepancies resolved by SK. This study used the Joanna Briggs Institute critical appraisal checklist for Randomized Controlled Trials (RCTs) and the Joanna Briggs Institute critical appraisal checklist for quasi‐experimental studies. This involved the three authors individually scoring each item on the checklist. Scoring was based on a three‐point scale: 1 (yes), −1 (no), and 0 (unclear), which reflected whether the studies met the specific criteria. The overall risk of bias for the studies was then calculated using percentage scores. Studies were categorized based on their quality: scores below 50% indicated low‐quality studies; those between 50% and 79% suggested moderate quality; and scores above 80% denoted high‐quality research.

### 2.4 Data Synthesis and Statistical Analysis

The team of authors engaged in a detailed discussion to categorize the interventions into five distinct groups: health education, information intervention, psychological intervention, exercise intervention, and mixed intervention. Given the unique nature of information interventions, which encompass a variety of frameworks, we opted to exclude them from the initial Network Meta‐Analysis (NMA). This analysis focused on the remaining four categories. Subsequently, based on the collected data, we sorted the information interventions into distinct frameworks. These included approaches centered on gain, norm, egoism, prosocial, health, narrative, high risk, low risk, guilt, positive, negative, threat, time, and combination.

Further, a Pairwise Meta‐Analysis (PMA) was conducted to evaluate the overall effectiveness of the interventions. This comprehensive analysis also included tests for heterogeneity and sensitivity, ensuring the robustness of our findings. Finally, the possibility of publication bias within the NMA results was rigorously assessed.

Pairwise meta‐analysis and network meta‐analysis were done using the meta package (Schwarzer 2007), metafor package (Viechtbauer 2010), and netmeta package (Schwarzer et al. 2015) in R language version 4.2.2. In PMA and NMA, since the data are continuous variables containing means and standard deviations and the measurement tools and methods used vary across health behaviors, SMD was used as the effect size, with 0.2 indicating a small effect, 0.5 indicating a medium effect, and 0.8 indicating a large effect (Borenstein et al. 2021). To mitigate the potential bias arising from baseline characteristics of study participants, we calculated the changes in mean and standard deviation values pre‐ and post‐intervention. This calculation was based on the Cochrane Handbook's approach, employing a correlation coefficient (*r* = 0.50) in the conversion formula for instances where studies did not report this coefficient directly. This method also applies to calculating endpoint values in the absence of baseline data, a practice supported by the Cochrane Handbook, which indicates that such data types can be combined for analysis. When standard deviation values were not available in the studies, corresponding formulas were used to transform existing data, such as SE values, 95% CI, medians, etc., and also to merge some data while ensuring less heterogeneity (Wan et al. 2014; Borenstein et al. 2021). The significance of the data was assessed based on whether the 95% CI included zero and the *p*‐value was less than 0.05.

In the PMA, the effect size was calculated using a random effects model, which assumes that differences in the studies arise not only from random errors but also from systematic errors and takes into account the heterogeneity in the studies and includes them in the calculations. Since the dependent variables in this paper are multiple behaviors and behavioral intentions and there are differences in the interventions, it can be inferred that there is strong heterogeneity between the studies, so a random effects model was used for the main effects test. For the heterogeneity test, the variability of effect sizes in the main study was assessed by Cochran's Q‐test results for significance, *I* ^*2*^, Tau^2^. A significant Q test result indicates significant heterogeneity, and *I* ^2^ > 75% indicates high heterogeneity, as well as a significant non‐zero Tau^2^ value, which also indicates high heterogeneity (Fu et al. 2011). In terms of stability of results, a sensitivity analysis was used for validation to exclude outliers from the study.

A random effects model was also used in the frequency NMA as well as SMD as an effect size, and each category of intervention will be compared with a control group, defined as the usual intervention or no intervention group relative to the intervention. We examined the potential effect modifiers that may exist in the study to test the transitivity assumption in the network meta‐analysis. We used node‐splitting analysis to test whether there is an inconsistency between direct and indirect evidence in the study. A network plot was used to visually depict the structure of the network and thus objectively evaluate the different interventions. Using the intervention group as a reference object, paired forest plots were created to evaluate the effectiveness of the different interventions for the intervention group. The effectiveness of different interventions was ranked using P‐score and the surface under the cumulative ranking (SUCRA), with P‐scores in the 0–1 range, with higher scores indicating better intervention effectiveness (Rücker and Schwarzer 2015; Shim et al. 2019). To further explore potential sources of heterogeneity and identify potential moderating effects, subgroup analyses and meta‐regression analyses were conducted. Finally, in terms of testing for publication bias, most of the methods used to assess publication bias in the PMA are not applicable to the NMA, so the comparison‐adjusted funnel plots were used to test for publication bias in the NMA (Salanti et al. 2014). This involved a qualitative examination of the distribution of effect sizes within the funnel plots to identify potential publication bias, followed by a quantitative assessment using Egger's regression test. Significant results from this test suggest a possible inclusion bias within the analyzed literature, indicating a predilection for publishing studies with positive or significant outcomes, which necessitates further discussion.

## 3 Results

**FIGURE 1:**
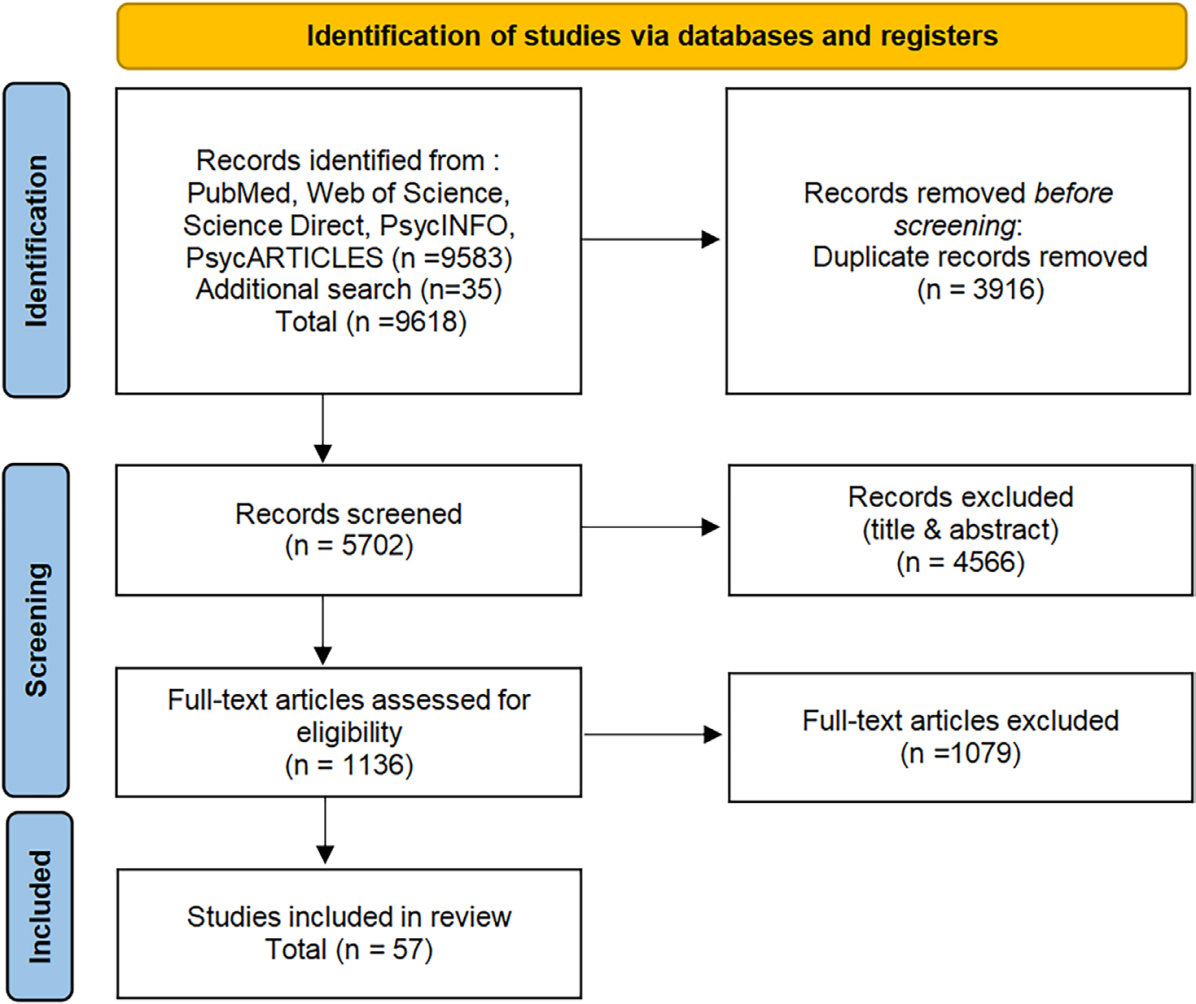
PRISMA flow diagram.

A total of 9618 studies were searched, of which 9583 were from the primary search database and 35 from the additional search (Figure 1). One thousand seventy‐eight articles were obtained in full text and further screened according to inclusion criteria, and 57 articles were retained. Fifty articles were retained for analysis in the PMA because 7 articles did not have a control group, and all articles were included in the NMA (K = 57). The PMA study included 44,799 participants with 144 effect sizes, and 57 articles contained a total of 47,264 participants.

For quality assessment, 29 RCT studies and 28 quasi‐experimental studies were included. Three RCTs had percentage scores below 50% versus three quasi‐experimental studies that had percentage scores below 50%. In addition, three RCTs and three quasi‐experimental studies had percentage scores above 80%, indicating good study quality, while the remaining 79.31% of studies showed moderate quality. Overall, the vast majority of studies were of moderate or above quality and, after discussion among several authors, we believe that the research quality is acceptable. Detailed scoring criteria and scoring are presented in Data S2, with additional interventions coded as specified in Data S1.

### 3.1 Pairwise Meta‐Analysis

**FIGURE 2:**
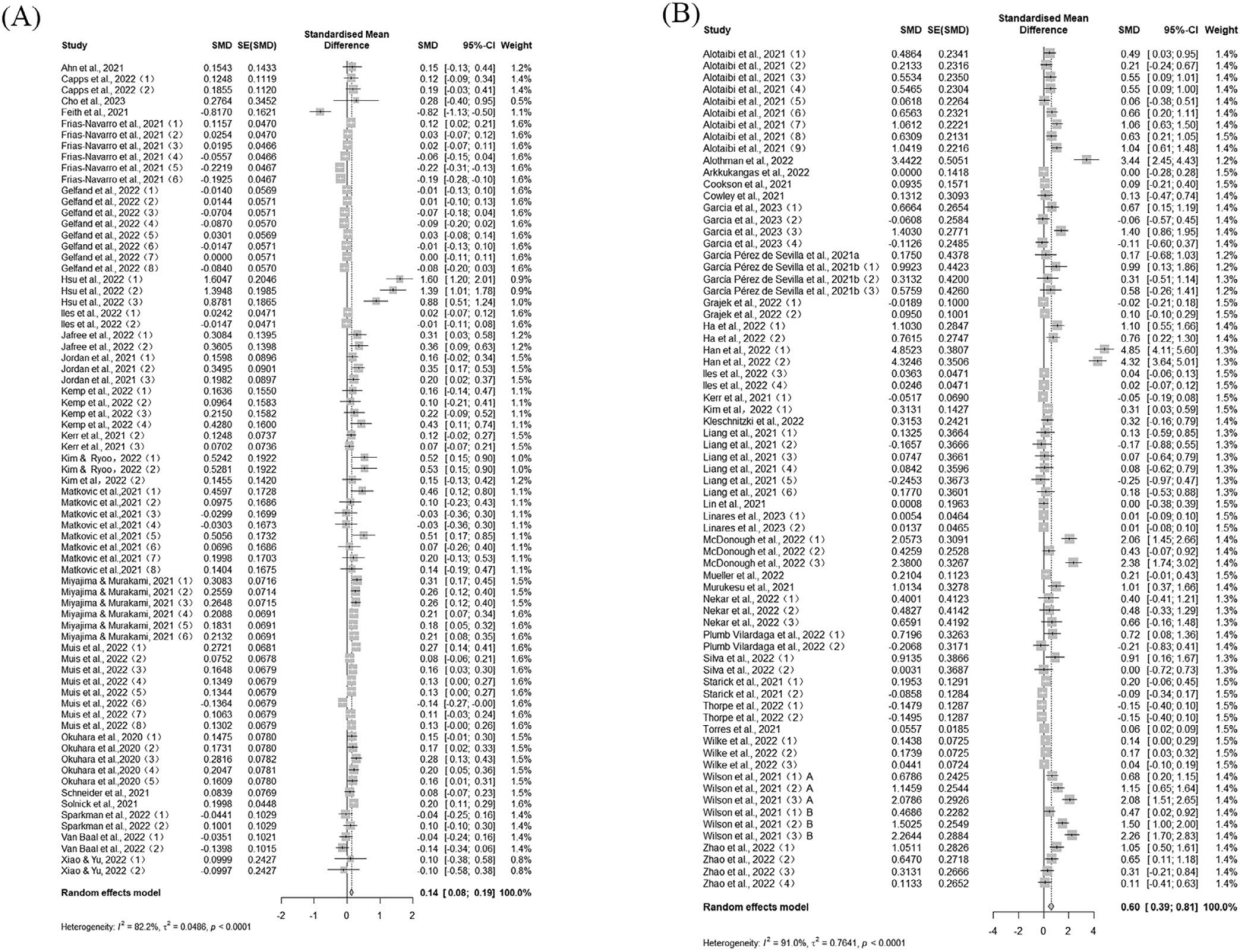
Pooled effect sizes for (A) preventive health behavior and (B) health‐promoting behavior.

On PHB (K = 73), the pooled effect of the intervention showed a significant but small effect (SMD = 0.14, *Z* = 5.05, 95% CI 0.09 to 0.20, *p* < 0.01), with reduced but still significant heterogeneity (*I* ^*2*^ = 82%, Tau^2^ = 0.05, *Q*[df(72)] = 399.4, *p* < 0.01). On HPB (K = 71), the intervention was significant (SMD = 0.60, *Z* = 5.52, 95% CI 0.39 to 0.81, *p* < 0.01) and heterogeneous (*I* ^*2*^ = 90.9%, Tau^2^ = 0.76, *Q*[df(70)] = 765.62, *p* < 0.01) (Figure 2). Sensitivity analysis for PHB showed little fluctuation in effect size after excluding one literature, and sensitivity analysis for HPB showed pooled effect size fluctuating from 0.52 to 0.61.

### 3.2 Network Meta‐Analysis

**FIGURE 3:**
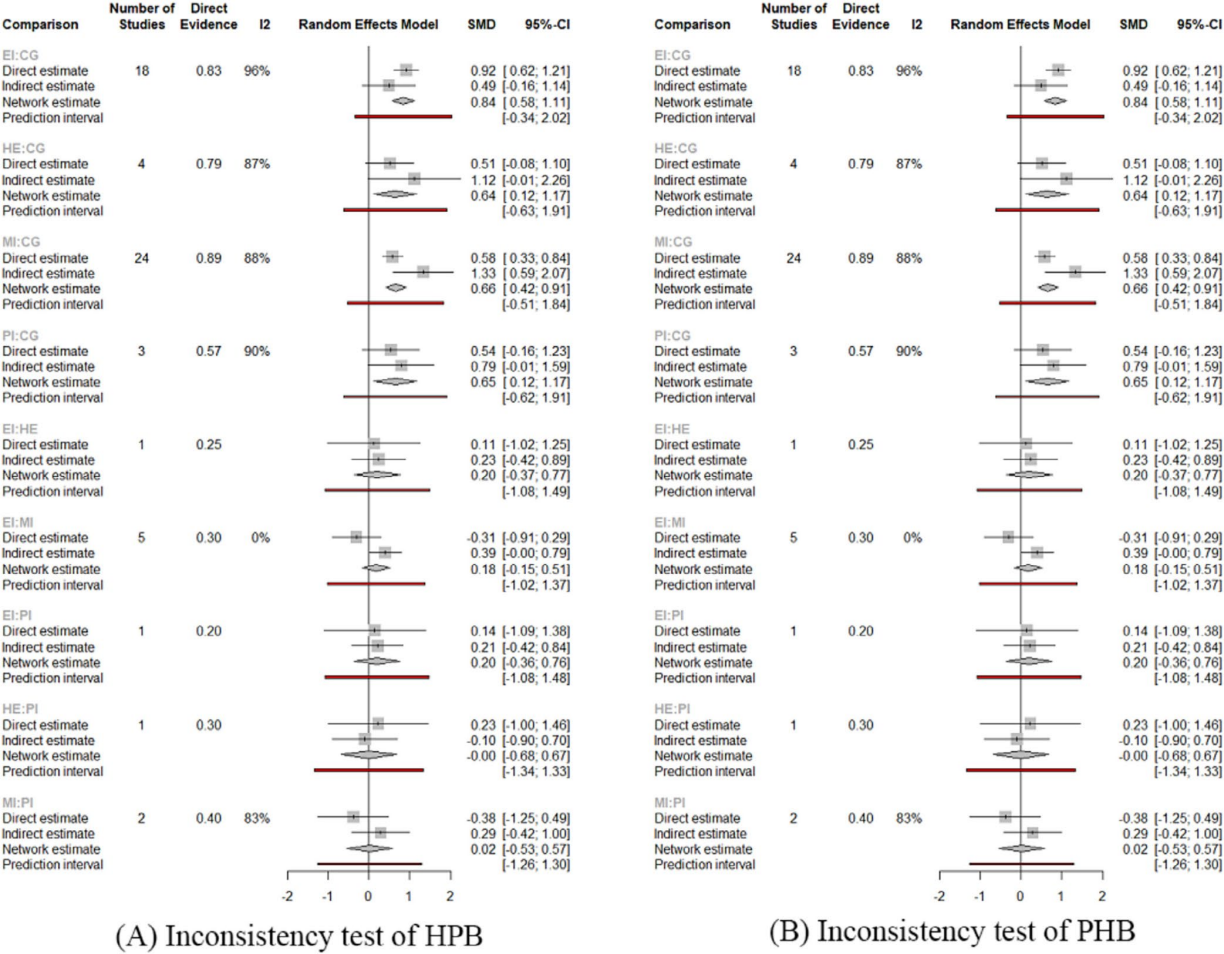
Inconsistency test for (A) preventive health behavior and (B) health‐promoting behavior.

For the transitivity assumption, Data S4 shows that there are no significant differences in the various characteristics of the study, including study design, study population, and intervention characteristics, although there are some differences to some extent. This homogeneity across studies, combined with the shared context of COVID‐19, suggests that the criteria for the transitivity assumption have been met, supporting the validity of conducting a network meta‐analysis. For the consistency assumption, the results (Figure 3) indicate that there is no significant difference between direct evidence and indirect evidence, and the consistency assumption can be satisfied.

**FIGURE 4:**
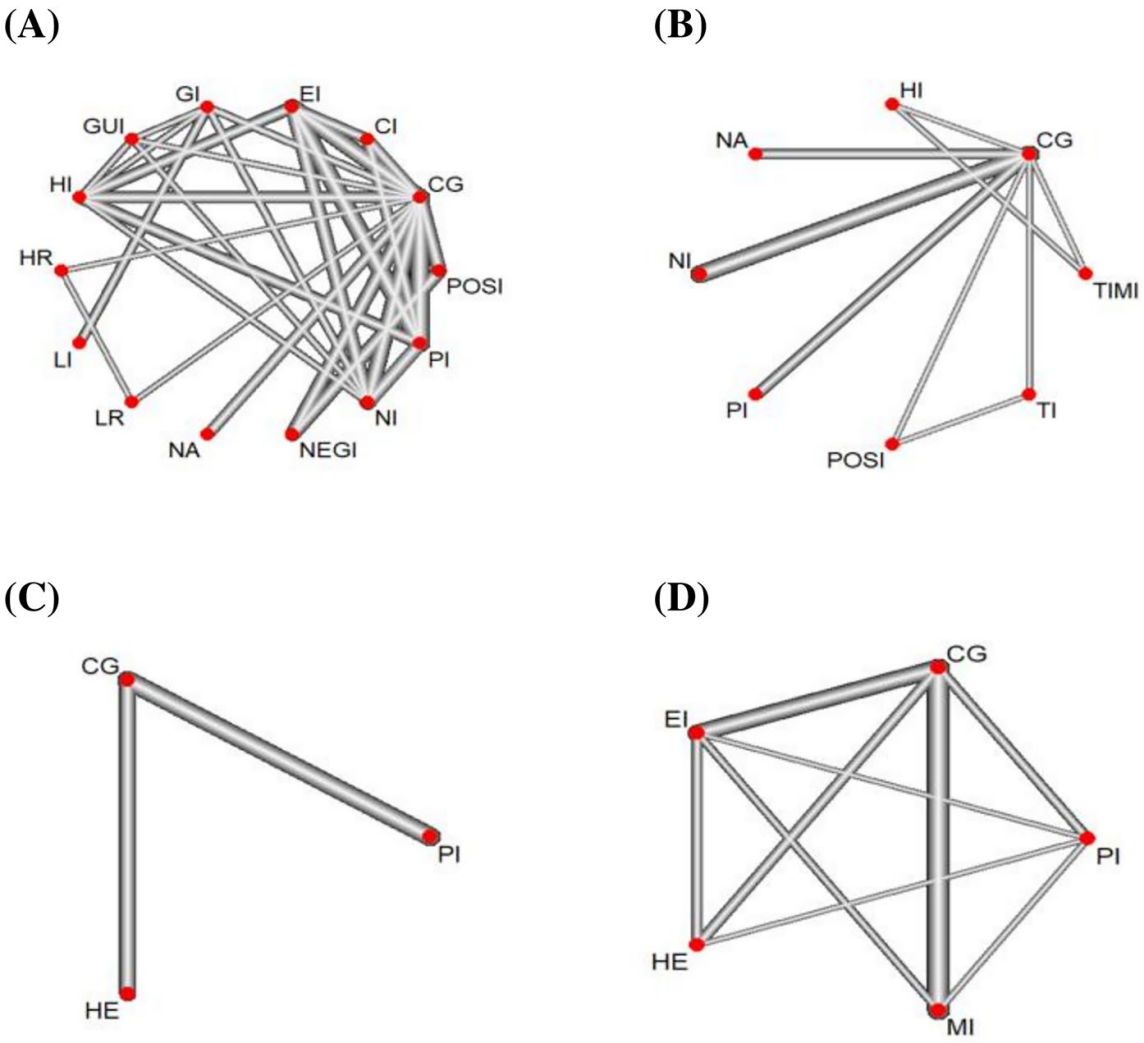
Network plot of various intervention measures. CG, control group; CI, combination information intervention; EI, egoism information intervention; EI, exercise intervention; GI, gain information intervention; GUI, guilt information intervention; HE, health education; HI, health information intervention; HR, high risk information intervention; LR, low risk information intervention; MI, mixed intervention; NA, narrative information intervention; NEGI, negative information intervention; NI, norm information intervention; PI, prosocial information intervention; PI, psychological intervention; POSI, positive information intervention; TI, threat information intervention; TIMI, time information intervention.

In all included studies, there were five types of interventions, including health education, information intervention, psychological intervention, exercise intervention, and mixed intervention. All network structures can be observed from the network plot (Figure 4). On PHB, 14 information frameworks were included. The low‐risk information framework (SMD = 0.25, 95% CI −0.16 to 0.66) was most likely to be the best intervention compared to the control group, with a P‐score of 0.77. For HPB, the prosocial information framework (SMD = 0.2, 95% CI 0.11 to 0.29) and the positive textual information framework (SMD = 0.2, 95% CI −0.05 to 0.45) were most likely to be the best interventions compared to the control group, with P‐scores of 0.92 and 0.87, respectively.

Next, we conducted NMAs for the remaining four types of interventions in terms of overall health behavior: health education, exercise intervention, psychological intervention, and combined intervention. On PHB, there were two types of interventions, health education and psychological intervention. The results showed that health education was likely to be the most effective intervention compared to the control group (SMD = 0.57, 95% CI 0.21 to 0.93) with a P‐score of 0.98. On HPB, four types of interventions existed: health education, exercise intervention, psychological intervention, and combined intervention, and the results showed that psychological intervention (SMD = 0.65, 95% CI 0.12 to 1.17), MI (SMD = 0.66, 95% CI 0.42 to 0.91), and health education (SMD = 0.64, 95% CI 0.12 to 1.17) had close effect sizes, while exercise intervention (SMD = 0.84, 95% CI 0.58 to 1.11) was the most effective intervention. Finally, the SUCRA probability ranking (see Tables 1, 2, 3, 4) also further shows the effectiveness of each type of intervention, with pairwise comparison results for each type of intervention.

**TABLE 1:** The intervention effect of information intervention on PHB.

	LR	POSI	HR	CI	HI	NI	EI	LI	PI	NEGI	GI	NA	GUI	CG
POSI	0.08 (−0.36, 0.52)													
HR	0.06 (−0.35, 0.47)	−0.02 (−0.45, 0.41)												
CI	0.13 (−0.31, 0.57)	0.05 (−0.17, 0.27)	0.07 (−0.37, 0.51)											
HI	0.13 (−0.30, 0.57)	0.05 (−0.16, 0.26)	0.07 (−0.36, 0.51)	0.00 (−0.20, 0.21)										
NI	0.14 (−0.28, 0.57)	0.07 (−0.12, 0.26)	0.09 (−0.34, 0.51)	0.02 (−0.17, 0.20)	0.01 (−0.16, 0.18)									
EI	0.15 (−0.27, 0.57)	0.07 (−0.11, 0.25)	0.09 (−0.33, 0.51)	0.02 (−0.14, 0.18)	0.02 (−0.14, 0.18)	0.01 (−0.13, 0.14)								
LI	0.19 (−0.32, 0.70)	0.11 (−0.23, 0.45)	0.13 (−0.38, 0.64)	0.06 (−0.28, 0.40)	0.06 (−0.27, 0.38)	0.04 (−0.27, 0.35)	0.04 (−0.28, 0.35)							
PI	0.19 (−0.23, 0.61)	0.11 (−0.07, 0.29)	0.13 (−0.29, 0.55)	0.06 (−0.09, 0.22)	0.06 (−0.10, 0.21)	0.04 (−0.08, 0.17)	0.04 (−0.06, 0.14)	0.00 (−0.31, 0.32)						
NEGI	0.20 (−0.24, 0.63)	0.12 (−0.03, 0.27)	0.14 (−0.29, 0.57)	0.07 (−0.15, 0.28)	0.07 (−0.14, 0.28)	0.05 (−0.14, 0.24)	0.05 (−0.13, 0.23)	0.01 (−0.33, 0.35)	0.01 (−0.17, 0.19)					
GI	0.22 (−0.23, 0.68)	0.14 (−0.11, 0.39)	0.16 (−0.29, 0.62)	0.09 (−0.15, 0.34)	0.09 (−0.13, 0.32)	0.08 (−0.13, 0.28)	0.07 (−0.14, 0.29)	0.04 (−0.19, 0.27)	0.03 (−0.18, 0.25)	0.03 (−0.23, 0.28)				
NA	0.25 (−0.25, 0.75)	0.17 (−0.15, 0.49)	0.19 (−0.30, 0.69)	0.12 (−0.20, 0.44)	0.12 (−0.20, 0.44)	0.10 (−0.20, 0.41)	0.10 (−0.20, 0.40)	0.06 (−0.35, 0.48)	0.06 (−0.24, 0.36)	0.05 (−0.27, 0.37)	0.03 (−0.32, 0.37)			
GUI	0.29 (−0.19, 0.77)	0.21 (−0.08, 0.50)	0.23 (−0.25, 0.71)	0.16 (−0.13, 0.45)	0.16 (−0.11, 0.42)	0.14 (−0.11, 0.40)	0.14 (−0.13, 0.40)	0.10 (−0.26, 0.46)	0.10 (−0.16, 0.36)	0.09 (−0.20, 0.38)	0.07 (−0.21, 0.34)	0.04 (−0.34, 0.42)		
CG	0.25 (−0.16, 0.66)	**0.17 (0.02, 0.32)**	0.19 (−0.22, 0.60)	0.12 (−0.03, 0.28)	0.12 (−0.03, 0.27)	0.10 (−0.01, 0.22)	0.10 (0.00, 0.20)	0.06 (−0.24, 0.37)	0.06 (−0.04, 0.16)	0.05 (−0.10, 0.20)	0.03 (−0.17, 0.23)	−0.00 (−0.28, 0.28)	−0.04 (−0.29, 0.21)	

*Note*: Bold values indicate comparatively larger effect estimates between interventions.

Abbreviations: CI, combination information intervention; CG, control group; EI, egoism information intervention; GI, gain information intervention; GUI, guilt information intervention; HI, health information intervention; HR, high risk information intervention; LR, low risk information intervention; NA, narrative information intervention; NEGI, negative information intervention; NI, norm information intervention; PI, prosocial information intervention.

**TABLE 2:** The intervention effect of information intervention on HPB.

	PI	POSI	NI	NA	CG	TI	HI	TIMI
POSI	0.00 (−0.27, 0.27)	POSI						
NI	**0.14 (0.05, 0.24)**	0.14 (−0.11, 0.40)						
NA	**0.17 (0.04, 0.30)**	0.17 (−0.10, 0.44)	0.03 (−0.07, 0.13)					
CG	**0.20 (0.11, 0.29)**	0.20 (−0.05, 0.45)	**0.06 (0.02, 0.09)**	0.03 (−0.06, 0.12)				
TI	**0.29 (0.02, 0.55)**	**0.29 (0.03, 0.54)**	0.14 (−0.11, 0.40)	0.12 (−0.15, 0.38)	0.09 (−0.16, 0.34)			
HI	**0.35 (0.08, 0.61)**	0.35 (−0.01, 0.70)	0.20 (−0.05, 0.46)	0.18 (−0.09, 0.44)	0.15 (−0.11, 0.40)	0.06 (−0.30, 0.41)		
TIMI	**0.35 (0.08, 0.61)**	0.35 (−0.01, 0.70)	0.20 (−0.05, 0.46)	0.18 (−0.09, 0.44)	0.15 (−0.11, 0.40)	0.06 (−0.30, 0.41)	0.00 (−0.25, 0.25)	

*Note*: Bold values indicate comparatively larger effect estimates between interventions.

Abbreviations: CG, control group; HI, health information intervention; NA, narrative information intervention; NI, norm information intervention; PI, prosocial information intervention; POSI, positive information intervention; TI, threat information intervention; TIMI, time information intervention.

**TABLE 3:** The intervention effect of intervention on PHB.

	HE	PI	CG
PI	0.40 (−0.07, 0.87)		
CG	**0.57 (0.21, 0.93)**	0.16 (−0.14, 0.47)	

*Note*: Bold values indicate comparatively larger effect estimates between interventions.

Abbreviations: CG, control group; HE, health education; PI, psychological intervention.

**TABLE 4:** The intervention effect of intervention on HPB.

	EI	PI	MI	HE	CG
PI	0.20 (−0.36, 0.76)				
MI	0.18 (−0.15, 0.51)	−0.02 (−0.57, 0.53)			
HE	0.20 (−0.37, 0.77)	0.00 (−0.67, 0.68)	0.02 (−0.55, 0.59)		
CG	**0.84 (0.58, 1.11)**	**0.65 (0.12, 1.17)**	**0.66 (0.42, 0.91)**	**0.64 (0.12, 1.17)**	

*Note*: Bold values indicate comparatively larger effect estimates between interventions.

Abbreviations: CG, control group; EI, exercise intervention; HE, health education; MI, mixed intervention; PI, psychological intervention.

### 3.3 Moderator Analysis and Sensitivity Analysis

We performed subgroup analyses based on country, study type, outcome, intervention form, and health behavior. Additionally, meta‐regression analyses were conducted with age, gender, and study quality as continuous moderators. The results indicated that these factors influenced the effect sizes to varying degrees (in Data S5).

Finally, sensitivity analyses were conducted. In the HPB group, the pooled effect size decreased from 0.60 to 0.47 after removing two outlier effect sizes from Han et al. (2022). In the PHB group, the pooled effect size decreased from 0.14 to 0.11 following the exclusion of two outlier effect sizes from Hsu et al. (2022) and one effect size from Feith et al. (2021). These findings suggest that although certain studies influenced the overall effect sizes, the results remained relatively robust.

### 3.4 Publication Bias

**FIGURE 5:**
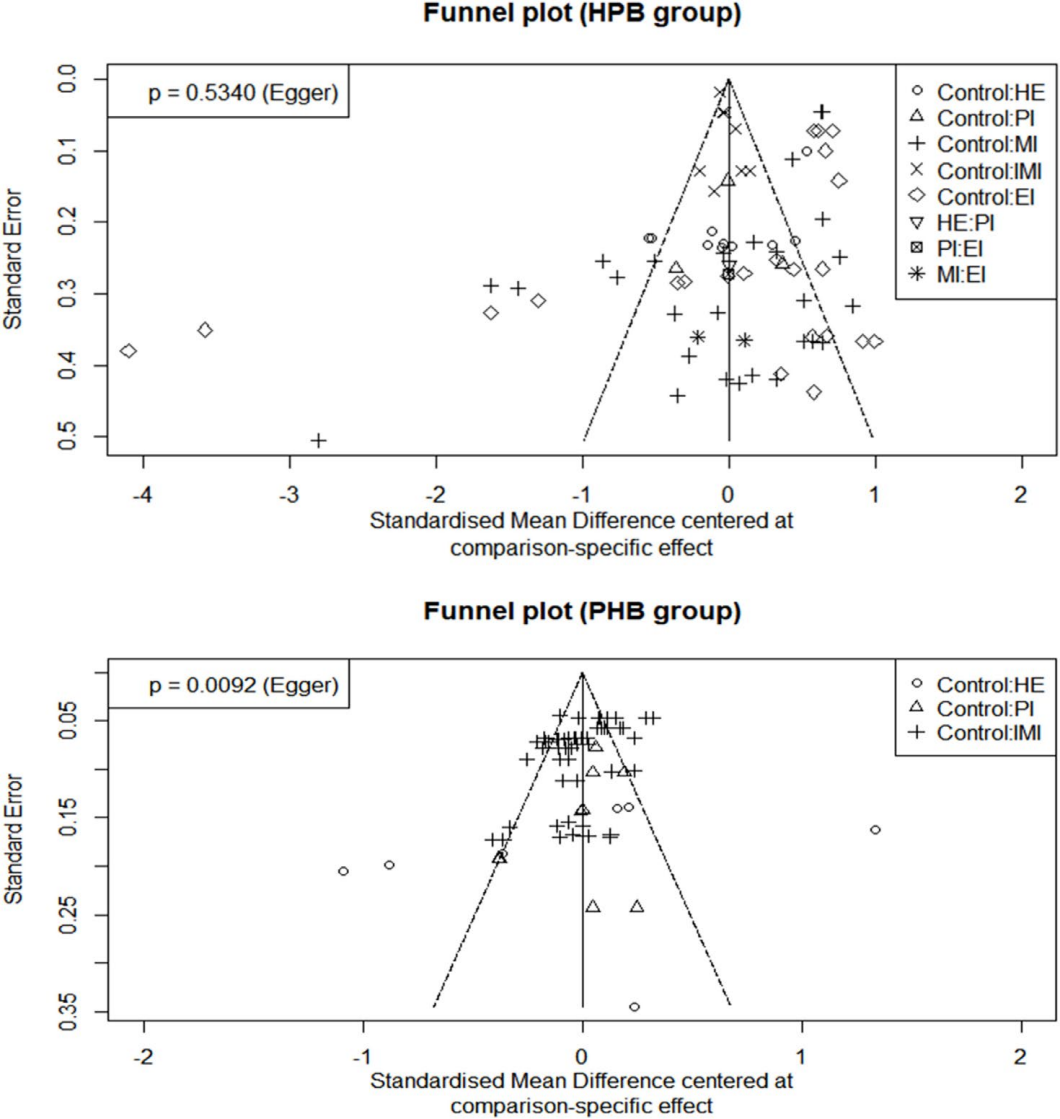
Funnel plot. CG, control group; EI, exercise intervention; HE, health education; IMI, information intervention; MI, mixed intervention; PI, psychological intervention.

Funnel plots for the HPB and PHB groups, along with Egger's regression test results (HPB: *p* = 0.534; PHB: *p* = 0.0092), indicate a potential risk of publication bias (Figure 5). Consequently, further publication bias tests were performed by distinguishing between information interventions and the other four intervention types (health education, exercise, mixed, and psychological interventions).

Four publication bias tests were conducted. The results of Egger's regression test showed that the publication bias of PHB studies under information intervention was significant (*p* < 0.01). The publication bias of HPB studies under information intervention was not significant (*p* = 0.25). The publication bias of PHB studies under health education, exercise intervention, mixed intervention, and psychological intervention was not significant (*p* = 0.42). The publication bias of HPB studies under health education, exercise intervention, mixed intervention, and psychological intervention was significant (*p* < 0.01).

## 4 Discussion

### 4.1 The Effectiveness of Health Behavior Intervention

In the study, a meta‐analysis of health behavior in the context of COVID‐19 was conducted using two meta‐analysis techniques, PMA and NMA. The overall intervention efficacy for PHB presented a small effect size (SMD = 0.14), while HPB presented a moderately large effect size (SMD = 0.60). These results are similar to previous studies (Krebs et al. 2010). This indicates that individuals in the intervention group demonstrated significantly improved health behaviors compared to those in the control group. The modest effect size observed for PHB interventions may be attributed to the physical and psychological side effects associated with these behaviors during the pandemic. Notable examples include breathing difficulties after mask‐wearing, skin reactions (Balestracci et al. 2023), and mental health challenges due to social distancing (Venkatesh and Edirappuli 2020), potentially diminishing the effectiveness of interventions targeting such behaviors. Conversely, the more substantial impact of HPB interventions can be explained by three factors. Firstly, HPB interventions, including those aimed at enhancing physical activity and nutrition, have been extensively researched, resulting in the development of systematic, scientifically grounded, and effective strategies that are readily applicable in COVID‐19 (Morgan et al. 2016). Secondly, unlike PHB, which often involves compulsory measures and involuntary actions, HPB stems from an individual's active desire to promote their health, encompassing activities like physical exercise that benefit both mental and physical well‐being (Codella et al. 2021). Lastly, HPB interventions are typically more conducive to long‐term implementation compared to PHB interventions, making them more acceptable to the public.

### 4.2 Interventions in Various Health Behaviors

It is important to note that, in the PMA, all types of findings should be taken with caution because we included studies with different types of variables and interventions. We also conducted a main effects test for the overall effectiveness of the information intervention, which showed a lower but still significant intervention effect of the information intervention (SMD = 0.09, 95% CI 0.05 to 0.12). Therefore, in the NMA, we separated informational interventions from other interventions and also distinguished between HPB and PHB. Firstly, we assessed the effectiveness of each informational intervention framework. In the PHB, effect sizes were higher for the low‐risk framework and the positive text framework, and significant results were found for the positive text framework. Rothman et al. (1993) suggested that positively framed messages would better promote preventive behavior relative to negatively framed messages, and it has been suggested that the emotions evoked by positive texts can promote people's ability to process information (Muis et al. 2015). In HPB, the positive textual information framework and the pro‐social information framework were the most effective interventions, and the pro‐social framework results were significant, suggesting that, similar to the findings of existing studies, pro‐social motivation can enhance the effectiveness of health behavior interventions (Betsch et al. 2017). Secondly, we examined the effectiveness of interventions other than informational interventions, and health education was superior to psychological interventions as the optimal intervention for PHB, possibly due to the higher applicability of health education interventions as a heuristic (Steckler et al. 1995) and the significant intervention effect in PHB during the pandemic (Keller et al. 2021). The reason for the exercise intervention being the most effective intervention in HPB may, on the one hand, come from the fact that the HPB included in this study are mostly physical activity, with variables such as exercise behavior. On the other hand, the effect of the exercise intervention may be effective not only for physical activity, but also for improving other health behaviors due to the self‐efficacy generated after exercise, thus leading to a higher intervention effect (Rhew et al. 2007). Regarding publication bias, Egger's regression test indicated partially significant results. This may be due to the moderate quality of most included studies, a scarcity of low‐bias risk articles, and the high heterogeneity among studies. These factors necessitate a cautious interpretation of the findings. Future research should take these considerations into account.

## 5 Limitations and Future Considerations

The limitations of the study are mainly as follows. Firstly, there are issues of publication bias and high heterogeneity. The reason for this comes from the fact that this paper incorporates both a quasi‐experimental design and an RCT design in the selection of the study design. Quasi‐experimental designs, while often more feasible in constrained settings such as the pandemic (Miller et al. 2020), may introduce bias. Limiting the meta‐analysis to a single study design could potentially reduce publication bias. High heterogeneity may raise doubts about the aggregation of research results, so in future research, we need to further investigate demographic characteristics, environmental factors, or other potential factors (including the limitations of the search databases) to understand the root causes of heterogeneity. Secondly, too many dependent variables were selected for this paper, especially in PHB. The diverse attitudes towards different health behaviors may account for varying intervention effects and potentially contribute to the noted publication bias. We found that many current studies do not clearly distinguish between the definition of preventive health behaviors and health‐promoting behaviors; therefore, future studies should distinguish and clarify the definition of each type of variable before conducting the study in order to subsequently develop corresponding interventions, which will also benefit the health management of the population. Finally, this paper does not directly compare the effects of information interventions with the other four types of interventions. The information framework intervention seems to be a better choice than the long‐term health behavior interventions (Balbo et al. 2015), which take a lot of effort and time. In the future, we should conduct a more detailed NMA research program using a refined and rigorous approach to further compare informational interventions with other interventions.

## 6 Conclusions

Overall, the interventions on health behaviors during COVID‐19 were effective. This study is the first to use a meta‐analytic approach to summarize health behavior interventions during COVID‐19 and to draw lessons for future pandemic response in the field of health behavior. Given the uncertain future impact of COVID‐19 or potential new epidemics on human life and well‐being, it becomes increasingly crucial to continue research and interventions in the field of health behavior to maximize the protection of human health.

## Ethics Statement

All procedures performed in studies involving human participants were in accordance with the ethical standards of the institutional and/or national research committee and with the 1964 Helsinki declaration and its later amendments or comparable ethical standards.

## Consent

Informed consent was obtained from all individual participants included in the study.

## Conflicts of Interest

The authors declare no conflicts of interest.

## Supporting information


**Data S1:** Supporting Information.

**Data S2:** Supporting Information.

**Data S3:** Supporting Information.

**Data S4:** Supporting Information.

**Data S5:** Supporting Information.

## Data Availability

The datasets generated during and/or analysed during the current study are available from the corresponding author on reasonable request.
