# Supplementary material for: Intervention Effectiveness of Health Behaviors During COVID‐19: A Systematic Review and a Network Meta‐Analysis
Source: Psych J. 2025 Sep 29;14(6):841–52. doi: 10.1002/pchj.70054 (PMC12702596; doi:10.1002/pchj.70054)
Supplement: Supplementary file 1 — Data S1: Supporting Information. [file PCHJ-14-841-s001.doc]

**Search strategy**

**PubMed**

("COVID-19"[MeSH Terms] OR "SARS-CoV-2"[MeSH Terms] OR "COVID"[Title/Abstract] OR "COVID-19"[Title/Abstract] OR "covid 19 pandemics"[Title/Abstract] OR "2019 ncov infection"[Title/Abstract] OR "covid 19 virus disease"[Title/Abstract] OR "2019 ncov disease"[Title/Abstract]) AND ("Health Behavior"[MeSH Terms] OR "behavior health"[Title/Abstract] OR "health related behavior"[Title/Abstract] OR "health behaviors"[Title/Abstract] OR "preventive behavior"[Title/Abstract] OR "protective behavior"[Title/Abstract] OR "self protective behavior"[Title/Abstract] OR "preventative behavior"[Title/Abstract] OR "vaccine behavior"[Title/Abstract] OR "checking behavior"[Title/Abstract] OR "Protective action"[Title/Abstract] OR "distance"[Title/Abstract] OR "Prevention"[Title/Abstract] OR "physical activity"[Title/Abstract] OR "preventative health behaviors"[Title/Abstract] OR "social distancing"[Title/Abstract] OR "hand washing"[Title/Abstract] OR "cleaning"[Title/Abstract] OR "disinfecting"[Title/Abstract] OR "face masks"[Title/Abstract] OR "mask"[Title/Abstract] OR "eye protection"[Title/Abstract] OR "wearing facemasks"[Title/Abstract] OR "defence"[Title/Abstract]) AND ("Randomized Controlled Trial"[Publication Type] OR "quasi-experimental"[Title/Abstract] OR "randomized controlled"[Title/Abstract] OR "randomized"[Title/Abstract] OR "random"[Title/Abstract] OR "experimental"[Title/Abstract] OR "Intervention"[Title/Abstract] OR "intervene"[Title/Abstract] OR "experiment"[Title/Abstract] OR "promoting"[Title/Abstract])

**web of science**

1: TS=(COVID-19) OR TS=(SARS-CoV-2)

2: TS=(Health Behavior OR Behavior, Health OR Health-Related Behavior OR Health Behaviors OR Preventive Behavior OR Protective Behavior OR Self-protective Behavior OR preventative behavior OR Vaccine Behavior OR Checking behavior OR Protective action OR disinfecting OR distance OR Prevention OR physical activity OR preventative health behaviors OR social distancing OR hand washing OR cleaning OR face masks OR mask OR eye protection OR wearing facemasks OR defence)

3: #1 AND #2

4: TS=(Randomized Controlled Trial OR quasi-experimental)

5: #3 AND #4

**Interpretation of coding**

**In information intervention**

CG=control group

GI=gain information intervention

NI=norm information intervention

EI=egoism information intervention

PI=prosocial information intervention

HI=health information intervention

NA=narrative information intervention

CI=combination information intervention

HR=high risk information intervention

LR=low risk information intervention

GUI=guilt information intervention

POSI=positive information intervention

NEGI=negative information intervention

TI=threat information intervention

TIMI=time information intervention

**In other intervention**

CG=control group

EI=exercise intervention

PI=psychological intervention

MI=mixed intervention

HE=health education
