# Supplementary material for: Intervention Effectiveness of Health Behaviors During COVID‐19: A Systematic Review and a Network Meta‐Analysis
Source: Psych J. 2025 Sep 29;14(6):841–52. doi: 10.1002/pchj.70054 (PMC12702596; doi:10.1002/pchj.70054)
Supplement: Supplementary file 2 — Data S2: Supporting Information. [file PCHJ-14-841-s002.doc]

Results of JBI critical appraisal checklist for randomized controlled trials

| Authors | True randomizati-on | Allocation to treatment groups concealed | Treatment groups similar at baseline | Participants blind to treatment assignment | Providers blind to treatment assignment | Outcome assess blind to treatment assignment | Treatment groups treated identically other than intervention of interest | Loss to follow up described | Intention-to-treat analysis | Outcomes measured in same way | Outcomes measured in reliable way | Appropriate statistical analysis used | Trial design appropriate | score | Percentage |
| --- | --- | --- | --- | --- | --- | --- | --- | --- | --- | --- | --- | --- | --- | --- | --- |
| Ahn et al., 2021 | Y | N | Y | N | N | Y | Y | Y | Y | Y | Y | Y | Y | 7 | 53.85% |
| Alotaibi et al., 2021 | Y | Y | Y | N | N | Y | U | U | Y | Y | Y | Y | Y | 7 | 53.85% |
| Alothman et al., 2022 | Y | Y | Y | N | N | Y | Y | N | Y | Y | Y | Y | Y | 7 | 53.85% |
| Arkkukangas et al.,2022 | Y | U | Y | N | N | Y | Y | Y | Y | Y | Y | Y | Y | 8 | 61.54% |
| Cookson et al., 2021 | Y | U | Y | N | N | Y | Y | Y | Y | Y | Y | Y | Y | 8 | 61.54% |
| Cowley et al., 2021 | Y | U | Y | N | N | Y | Y | N | Y | Y | Y | Y | Y | 7 | 53.85% |
| Feith et al., 2021 | Y | N/A | Y | U | N | Y | Y | N | Y | Y | Y | Y | Y | 7 | 58.33% |
| García Pérez de Sevilla et al., 2021a | Y | U | Y | U | N | Y | Y | Y | Y | Y | Y | Y | Y | 9 | 69.23% |
| García Pérez de Sevilla et al., 2021b | Y | U | Y | U | N | Y | U | Y | Y | Y | Y | Y | Y | 8 | 61.54% |
| Garcia et al., 2023 | Y | Y | Y | N | N | Y | Y | Y | Y | Y | Y | Y | Y | 9 | 69.23% |
| Grajek et al., 2022 | Y | Y | Y | N | N | Y | Y | N | Y | Y | Y | Y | Y | 7 | 53.85% |
| Granet et al., 2022 | Y | Y | Y | N | N | Y | Y | Y | Y | Y | Y | Y | Y | 9 | 69.23% |
| Ha et al., 2022 | Y | U | Y | N | N | Y | Y | N | Y | Y | Y | Y | Y | 6 | 46.15% |
| Hsu et al.,2022 | Y | U | Y | N | N | Y | Y | Y | Y | Y | Y | Y | Y | 8 | 61.54% |
| Jafree et al., 2022 | Y | U | Y | N | N | Y | Y | N | Y | Y | Y | Y | Y | 6 | 46.15% |
| Kim et al., 2022 | Y | Y | U | Y | U | Y | Y | N/A | Y | Y | Y | Y | Y | 10 | 83.33% |
| Laird et al., 2022 | Y | Y | Y | N | N | Y | Y | Y | Y | Y | Y | Y | Y | 9 | 69.23% |
| Liang et al.,2021 | Y | U | Y | N | N | Y | Y | N | Y | Y | Y | Y | Y | 6 | 46.15% |
| Lin et al.,2021 | Y | Y | Y | Y | N | Y | Y | Y | Y | Y | Y | Y | Y | 11 | 84.62% |
| McDonough et al.,2022 | Y | Y | Y | N | N | Y | Y | N | Y | Y | Y | Y | Y | 7 | 53.85% |
| Mueller et al.,2022 | Y | Y | Y | N | N | Y | Y | Y | Y | Y | Y | Y | Y | 9 | 69.23% |
| Murukesu et al.,2021 | Y | U | Y | U | N | Y | Y | N | Y | Y | Y | Y | Y | 7 | 53.85% |
| Nekar et al.,2022 | Y | N | Y | N | N | Y | Y | Y | Y | Y | Y | Y | Y | 7 | 53.85% |
| Plumb Vilardaga et al.,2022 | Y | U | Y | U | N | Y | U | Y | Y | Y | Y | Y | Y | 8 | 61.54% |
| Schneider et al.,2021 | Y | Y | Y | U | N | Y | Y | N | Y | Y | Y | Y | Y | 8 | 61.54% |
| Seixas et al.,2022 | Y | Y | Y | Y | Y | Y | Y | Y | Y | Y | U | Y | Y | 12 | 92.31% |
| Silva et al.,2022 | Y | Y | Y | U | N | Y | Y | Y | Y | Y | Y | Y | Y | 10 | 76.92% |
| Torres et al., 2021 | Y | U | Y | U | N | Y | U | Y | Y | Y | Y | Y | Y | 8 | 61.54% |
| Wilke et al.,2022 | Y | Y | Y | U | N | Y | Y | N | Y | Y | Y | Y | Y | 8 | 61.54% |
| Zhao et al.,2022 | Y | Y | Y | N | N | Y | Y | N | Y | Y | Y | Y | Y | 7 | 53.85% |

Results of JBI critical appraisal checklist for quasi-experimental

| Authors | Outcome measured as effect of the potential cause | Treatment groups similar at baseline | Treatment groups treated identically other than intervention of interest | Control group | Multiple pre- and post-intervention measurements | Loss to follow up described | Outcomes measured in same way | Outcomes measured in reliable way | Appropriate statistical analysis used | Score | Percentage |
| --- | --- | --- | --- | --- | --- | --- | --- | --- | --- | --- | --- |
| Capps et al.,2022 | Y | Y | Y | N | N | N/A | Y | Y | Y | 4 | 50% |
| Ceylan & Hayran, 2021 | Y | Y | Y | N/A | N | N/A | Y | Y | Y | 5 | 71.43% |
| Cho et al., 2023 | Y | Y | Y | Y | Y | N/A | Y | Y | Y | 8 | 100% |
| Frias-Navarro et al., 2021 | Y | Y | Y | Y | N | N/A | Y | Y | Y | 6 | 75% |
| Friedman et al., 2022 | Y | Y | Y | N | Y | Y | Y | Y | Y | 7 | 77.7% |
| Gelfand et al., 2022 | Y | Y | Y | Y | N | N/A | Y | Y | Y | 6 | 75% |
| Han et al.,2022 | Y | Y | Y | Y | Y | Y | Y | Y | Y | 9 | 100% |
| Iles et al., 2022 | Y | Y | Y | Y | N | N/A | Y | Y | Y | 6 | 75% |
| Jordan et al.,2021 | Y | Y | N | Y | N | N/A | Y | U | Y | 3 | 37.5% |
| Kemp et al.,2021 | Y | Y | Y | Y | N | N/A | Y | Y | Y | 6 | 75% |
| Kerr et al.,2021 | Y | Y | N | Y | N | N/A | Y | Y | Y | 4 | 50% |
| Kim & Ryoo, 2022 | Y | Y | Y | N | N | N/A | Y | Y | Y | 4 | 50% |
| Kleschnitzki et al., 2022 | Y | Y | N | Y | N | N/A | Y | Y | Y | 4 | 50% |
| Linares et al., 2023 | Y | Y | N/A | Y | Y | N/A | Y | Y | Y | 7 | 100% |
| Matkovic et al., 2021 | Y | Y | Y | N | N | N/A | Y | Y | Y | 4 | 50% |
| Miyajima & Murakami, 2021 | Y | Y | N | Y | N | N/A | Y | Y | Y | 4 | 50% |
| Muis et al.,2022 | Y | Y | N | Y | N | Y | Y | Y | Y | 5 | 55.5% |
| Neumer et al., 2022 | Y | Y | N/A | N | N | N/A | Y | Y | Y | 4 | 57.14% |
| Okuhara et al., 2020 | Y | Y | N/A | Y | N | N/A | Y | Y | Y | 5 | 71.43% |
| Peng et al., 2022 | Y | Y | N/A | N | N | N/A | Y | Y | Y | 3 | 42.86% |
| Solnick et al., 2021 | Y | Y | Y | N | N | N/A | Y | Y | Y | 4 | 50% |
| Sparkman et al., 2022 | Y | Y | Y | N | N | N/A | Y | Y | Y | 4 | 50% |
| Starick et al., 2021 | Y | Y | N/A | N | N | N/A | Y | Y | Y | 3 | 42.86% |
| Thorpe et al., 2022 | Y | Y | N | Y | N | N/A | Y | Y | Y | 4 | 50% |
| van Baal et al., 2022 | Y | Y | Y | N | N | N/A | Y | Y | Y | 4 | 50% |
| Wilson et al., 2021 A | Y | Y | N | Y | Y | Y | Y | Y | Y | 7 | 77.7% |
| Wilson et al., 2021 B | Y | Y | N | Y | Y | N/A | Y | Y | Y | 6 | 75% |
| Xiao & Yu, 2022 | Y | Y | Y | N | N | N/A | Y | Y | Y | 4 | 50% |
