# Supplementary material for: Intervention Effectiveness of Health Behaviors During COVID‐19: A Systematic Review and a Network Meta‐Analysis
Source: Psych J. 2025 Sep 29;14(6):841–52. doi: 10.1002/pchj.70054 (PMC12702596; doi:10.1002/pchj.70054)
Supplement: Supplementary file 3 — Data S3: Supporting Information. [file PCHJ-14-841-s005.doc]

Literature included in meta-analysis

*Ahn, J. N., Hu, D., & Vega, M. (2021). Changing pace: Using implementation intentions to enhance social distancing behavior during the COVID-19 pandemic. *Journal of Experimental Psychology: Applied, 27*(4), 762.https://doi.org/10.1037/xap0000385

*Alotaibi, N., Al-Sayegh, N., Nadar, M., Shayea, A., Allafi, A., & Almari, M. (2021). Investigation of Health Science Students' Knowledge Regarding Healthy Lifestyle Promotion During the Spread of COVID-19 Pandemic: A Randomized Controlled Trial. *Frontiers in Public Health, 9*, 774678.https://doi.org/10.3389/fpubh.2021.774678

*Alothman, S. A., Alshehri, M. M., Almasud, A. A., Aljubairi, M. S., Alrashed, I., Abu Shaphe, M., & Alghannam, A. F. (2022, December). Virtual Behavioral Intervention to Promote Healthy Lifestyle Behaviors: A Feasibility RCT during COVID-19 Pandemic. *In Healthcare*(Vol. 11, No. 1, p. 91). MDPI.https://doi.org/10.3389/10.3390/healthcare11010091

*Arkkukangas, M., Strömqvist Bååthe, K., Ekholm, A., & Tonkonogi, M. (2022). High challenge exercise and learning safe landing strategies among community-dwelling older adults: a randomized controlled trial. *International Journal of Environmental Research and Public Health, 19*(12), 7370.https://doi.org/10.3390/ijerph19127370

*Capps, K. P., Updegraff, J. A., Foust, J. L., O'Brien, A. G., & Taber, J. M. (2022). Field experiment of signs promoting hand hygiene during the COVID-19 pandemic. *Health Psychology, 41*(11), 826*.*https://doi.org/10.1037/hea0001211

*Ceylan, M., & Hayran, C. (2021). Message framing effects on individuals' social distancing and helping behavior during the COVID-19 pandemic.*Frontiers in Psychology, 12,* 579164.https://doi.org/10.3389/fpsyg.2021.579164

*Cho, Y. H., Cho, J., & Cho, O. H. (2023). Effects of self-management education on COVID-19 prevention in patients undergoing hemodialysis. *Heliyon*, e12931.https://doi.org/10.1016/j.heliyon.2023.e12931

*Cookson, D., Jolley, D., Dempsey, R. C., & Povey, R. (2021). A social norms approach intervention to address misperceptions of anti-vaccine conspiracy beliefs amongst UK parents.*PLOS One, 16*(11), e0258985.https://doi.org/10.1371/journal.pone.0258985

*Cowley, E. S., Watson, P. M., Foweather, L., Belton, S., Mansfield, C., Whitcomb-Khan, G., ... & Wagenmakers, A. J. (2021). Formative evaluation of a home-based physical activity intervention for adolescent girls—The HERizon project: A randomised controlled trial. *Children, 8*(2), 76.https://doi.org/10.3390/children8020076

*Feith, H. J., Lehotsky, Á., Gézsi, A., Lukács, J. Á., Gradvohl, E., & Falus, A. (2021). Egészségpedagógiai tapasztalatok a gyermekek kézmosásának oktatásában; higiénés nevelés a világjárványban. *Orvosi Hetilap, 162*(46), 1842-1847.https://doi.org/10.1556/650.2021.32382

*Frias-Navarro, D., Pascual-Soler, M., Berrios-Riquelme, J., Gomez-Frias, R., & Caamaño-Rocha, L. (2021). COVID–19. Effect of moral messages to persuade the population to stay at home in Spain, Chile, and Colombia.*The Spanish Journal of Psychology, 24*, e42.https://doi.org/10.1017/SJP.2021.39

*Friedman, K., Marenus, M. W., Murray, A., Cahuas, A., Ottensoser, H., Sanowski, J., & Chen, W. (2022). Enhancing physical activity and psychological well-being in college students during COVID-19 through WeActive and WeMindful interventions. *International Journal of Environmental Research and Public Health, 19*(7), 4144.https://doi.org/10.3390/ijerph19074144

*García Pérez de Sevilla, G., Barceló Guido, O., De la Cruz, M. D. L. P., Blanco Fernández, A., Alejo, L. B., Montero Martínez, M., & Pérez-Ruiz, M. (2021). Adherence to a lifestyle exercise and nutrition intervention in university employees during the COVID-19 pandemic: A randomized controlled trial.*International Journal of Environmental Research and Public Health, 18*(14),7510.https://doi.org/10.3390/ijerph18147510

*García Pérez de Sevilla, G., Barceló Guido, O., De la Cruz, M. D. L. P., Fernández, A. B., Alejo, L. B., Ramírez Goercke, M. I., & Pérez-Ruiz, M. (2021). Remotely supervised exercise during the COVID-19 pandemic versus in-person-supervised exercise in achieving long-term adherence to a healthy lifestyle. *International Journal of Environmental Research and Public Health, 18*(22), 12198.https://doi.org/10.3390/ijerph182212198

*Garcia, A., Yáñez, A. M., Bennasar-Veny, M., Navarro, C., Salva, J., Ibarra, O., ... & Garcia-Toro, M. (2023). Efficacy of an adjuvant non-face-to-face multimodal lifestyle modification program for patients with treatment-resistant major depression: A randomized controlled trial. *Psychiatry Research, 319*, 114975.https://doi.org/10.1016/j.psychres.2022.114975

*Gelfand, M., Li, R., Stamkou, E., Pieper, D., Denison, E., Fernandez, J., ... & Dimant, E. (2022). Persuading republicans and democrats to comply with mask wearing: An intervention tournament. *Journal of Experimental Social Psychology, 101*, 104299.https://doi.org/10.1016/j.jesp.2022.104299

*Grajek, M., Gdańska, A., Krupa-Kotara, K., Głogowska-Ligus, J., & Kobza, J. (2022). Global Self-Esteem, Physical Activity, and Body

Composition Changes Following a 12-Week Dietary and Physical Activity Intervention in Older Women.*International Journal of Environmental Research and Public Health, 19*(20), 13220.https://doi.org/10.3390/ijerph192013220

*Ha, Y., Lee, S. H., Lee, D. H., Kang, Y. H., Choi, W., & An, J. (2022). Effectiveness of a mobile wellness program for nurses with rotating shifts during COVID-19 pandemic: a pilot cluster-randomized trial. *International Journal of Environmental Research and Public Health, 19*(2), 1014.https://doi.org/10.3390/ijerph19021014

*Han, X., Tian, Z., Zhao, M., & Zhou, Z. (2022). An Intervention Pattern of Family Parent-Child Physical Activity Based on a Smartphone App for Preschool Children during COVID-19. *BioMed Research International, 2022*(1), 2777079*.*https://doi.org/10.1155/2022/2777079

*Hsu, Y. M., Chang, T. S., Chu, C. L., Hung, S. W., Wu, C. J., Yeh, T. P., & Wang, J. Y. (2022, June). Effectiveness of Multimedia-Based Learning on the Improvement of Knowledge, Attitude, and Behavioral Intention toward COVID-19 Prevention among Nurse Aides in Taiwan: A Parallel-Interventional Study. *In Healthcare* (Vol. 10, No. 7, p. 1206). MDPI.https://doi.org/10.3390/healthcare10071206

*Iles, I. A., Gaysynsky, A., & Sylvia Chou, W. Y. (2022). Effects of Narrative Messages on Key COVID-19 Protective Responses: Findings From a Randomized Online Experiment. *American Journal of Health Promotion, 36*(6), 934-947.https://doi.org/10.1177/08901171221075612

*Jafree, S. R., Zakar, R., Rafiq, N., Javed, A., Durrani, R. R., Burhan, S. K., ... & Fischer, F. (2022). WhatsApp-Delivered intervention for continued learning for nurses in Pakistan during the COVID-19 pandemic: results of a randomized-controlled trial. *Frontiers in Public Health, 10*, 739761.https://doi.org/10.3389/fpubh.2022.739761

*Jordan, J. J., Yoeli, E., & Rand, D. G. (2021). Don’t get it or don’t spread it: Comparing self-interested versus prosocial motivations for COVID-19 prevention behaviors. *Scientific Reports, 11*(1), 20222.https://doi.org/10.1038/s41598-021-97617-5

*Kemp, D., King, A. J., Upshaw, S. J., Mackert, M., & Jensen, J. D. (2022). Applying harm reduction to COVID-19 prevention: the influence of moderation messages and risk infographics. *Patient Education and Counseling, 105*(2), 269-276.https://doi.org/10.1016/j.pec.2021.09.006

*Kerr, J. R., Freeman, A. L., Marteau, T. M., & van der Linden, S. (2021). Effect of information about COVID-19 vaccine effectiveness and side effects on behavioural intentions: two online experiments.*Vaccines, 9*(4), 379.https://doi.org/10.3390/vaccines9040379

*Kim, S., Gollwitzer, P. M., & Oettingen, G. (2022). Mental contrasting of a negative future facilitates COVID-19 preventative behaviors: two randomized controlled trials. *Psychology & Health*, 1-23.https://doi.org/10.1080/08870446.2022.2060978

*Kim, W., & Ryoo, Y. (2022). Hypocrisy induction: Using chatbots to promote covid-19 social distancing.*Cyberpsychology, Behavior, and Social Networking, 25*(1), 27-36.https://doi.org/10.1089/cyber.2021.0057

*Kleschnitzki, J. M., Grossmann, I., Beyer, R., & Beyer, L. (2022). Modification in the Motor Skills of Seniors in Care Homes Using Serious Games and the Impact of COVID-19: Field Study.*JMIR Serious Games, 10*(2), e36768.https://doi.org/10.2196/36768

*Laird, B., Puzia, M., Larkey, L., Ehlers, D., & Huberty, J. (2022). A Mobile App for Stress Management in Middle-Aged Men and Women (Calm): Feasibility Randomized Controlled Trial. *JMIR Formative Research, 6*(5), e30294.https://doi.org/10.2196/30294

*Liang, I. J., Perkin, O. J., McGuigan, P. M., Thompson, D., & Western, M. J. (2021). Feasibility and acceptability of home-based exercise snacking and tai chi snacking delivered remotely to self-isolating older adults during COVID-19. *Journal of Aging and Physical Activity, 30*(1), 33-43.https://doi.org/10.1123/japa.2020-0391

*Lin, C. L., Huang, L. C., Chang, Y. T., Chen, R. Y., & Yang, S. H. (2021). Under COVID-19 Pandemic: A Quasi-Experimental Trial of Observation on Diabetes Patients' Health Behavior Affected by the Pandemic From a Coaching Intervention Program. *Frontiers in Public Health*, *9*, 580032.https://doi.org/10.3389/fpubh.2021.580032

*Linares, A., Plank, K., Hewawitharana, S. C., & Woodward-Lopez, G. The impact of SNAP-Ed interventions on California students’ diet and physical activity during COVID-19. *Public Health Nutrition*, 1-28.https://doi.org/10.1017/S1368980023000137

*Matkovic, J., Clemens, K. S., Faasse, K., & Geers, A. L. (2021). Handwashing message type predicts behavioral intentions in the United States at the beginning of the global COVID-19 pandemic.*Frontiers in public health*,*9,* 583491. https://doi.org/10.3389/fpubh.2021.583491

*McDonough, D. J., Helgeson, M. A., Liu, W., & Gao, Z. (2022). Effects of a remote, YouTube-delivered exercise intervention on young adults’ physical activity, sedentary behavior, and sleep during the COVID-19 pandemic: Randomized controlled trial.*Journal of Sport and Health Science, 11*(2), 145-156.https://doi.org/10.1016/j.jshs.2021.07.009

*Miyajima, T., & Murakami, F. (2021). Self-interested framed and prosocially framed messaging can equally promote COVID-19 prevention intention: A replication and extension of Jordan et al.’s study (2020) in the Japanese context. *Frontiers in Psychology*,*12*, 605059.https://doi.org/10.3389/fpsyg.2021.605059

*Mueller, J., Richards, R., Jones, R. A., Whittle, F., Woolston, J., Stubbings, M., ... & Ahern, A. L. (2022). Supporting Weight Management during COVID-19: A Randomized Controlled Trial of a Web-Based, ACT-Based, Guided Self-Help Intervention. *Obesity Facts, 15*(4), 550-559.https://doi.org/10.1159/000524031

*Muis, K. R., Sinatra, G. M., Pekrun, R., Kendeou, P., Mason, L., Jacobs, N., ... & Losenno, K. M. (2022). Flattening the COVID-19 curve: Emotions mediate the effects of a persuasive message on preventive action. *Frontiers in Psychology*,*13,* 1047241.https://doi.org/10.3389/fpsyg.2022.1047241

*Murukesu, R. R., Singh, D. K. A., Shahar, S., & Subramaniam, P. (2021). Physical activity patterns, psychosocial well-being and coping strategies among older persons with cognitive frailty of the “WE-RISE” trial throughout the COVID-19 movement control order. *Clinical Interventions in Aging,* 415-429.https://doi.org/10.2147/CIA.S290851

*Nekar, D. M., Kang, H. Y., & Yu, J. H. (2022). Improvements of Physical Activity Performance and Motivation in Adult Men through Augmented Reality Approach: A Randomized Controlled Trial. *Journal of Environmental and Public Health*, *2022*(1), 3050424.https://doi.org/10.1155/2022/3050424

*Neumer, A., Schweizer, T., Bogdanić, V., Boecker, L., & Loschelder, D. D. (2022). How health message framing and targets affect distancing during the COVID-19 pandemic.*Health Psychology, 41*(9), 630.https://doi.org/10.1037/hea0001203

*Okuhara, T., Okada, H., & Kiuchi, T. (2020). Examining persuasive message type to encourage staying at home during the COVID-19 pandemic and social lockdown: A randomized controlled study in Japan.*Patient Education and Counseling, 103*(12), 2588-2593.https://doi.org/10.1016/j.pec.2020.08.016

*Peng, L., Jiang, H., Guo, Y., & Hu, D. (2022). Effect of information framing on wearing masks during the COVID-19 pandemic: Interaction with social norms and information credibility. *Frontiers in Public Health,* *10*, 811792.https://doi.org/10.3389/fpubh.2022.811792

*Plumb Vilardaga, J. C., Kelleher, S. A., Diachina, A., Riley, J., & Somers, T. J. (2022). Linking physical activity to personal values: feasibility and acceptability randomized pilot of a behavioral intervention for older adults with osteoarthritis pain. *Pilot and Feasibility Studies*, *8*(1), 164.https://doi.org/10.1186/s40814-022-01121-0

*Schneider, C. R., Freeman, A. L., Spiegelhalter, D., & van der Linden, S. (2021). The effects of quality of evidence communication on perception of public health information about COVID-19: Two randomised controlled trials.*PlOS One, 16*(11), e0259048.https://doi.org/10.1371/journal.pone.0259048

*Seixas, M. B., Pereira, D. A. G., de Melo Ghisi, G. L., Batalha, A. P. D. B., de Oliveira Santos, C. V., Ponciano, I. C., ... & da Silva, L. P. (2022). Exercise and Lifestyle Education program for Brazilians living with prediabetes and diabetes: A pilot randomized trial.*Diabetes & Metabolic Syndrome: Clinical Research & Reviews, 16*(10), 102614.https://doi.org/10.1016/j.dsx.2022.102614

*Silva, F. M., Duarte-Mendes, P., Carvalho, E., Soares, C. M., Farinha, C., Serrano, J., ... & Ferreira, J. P. (2022). Effects of combined training during the COVID-19 pandemic on metabolic health and quality of life in sedentary workers: A randomized controlled study.*Frontiers in Public Health, 10*,1040714.https://doi.org/10.3389/fpubh.2022.1040714

*Solnick, R. E., Chao, G., Ross, R. D., Kraft‐Todd, G. T., & Kocher, K. E. (2021). Emergency physicians and personal narratives improve the perceived effectiveness of COVID‐19 public health recommendations on social media: a randomized experiment. *Academic Emergency Medicine, 28*(2), 172-183.https://doi.org/10.1111/acem.14188

*Sparkman, D. J., Kleive, K., & Ngu, E. (2022). Does activating the human identity improve health-related behaviors during COVID-19?: A social identity approach. *Frontiers in Psychology, 13*, 810805.https://doi.org/10.3389/fpsyg.2022.810805

*Starick, E., Montemarano, V., & Cassin, S. E. (2021). Coping during COVID-19: The Impact of Cognitive Appraisal on Problem Orientation, Coping Behaviors, Body Image, and Perceptions of Eating Behaviors and Physical Activity during the Pandemic.*International Journal of Environmental Research and Public Health, 18*(21), 11305.https://doi.org/10.3390/ijerph182111305

*Thorpe, A., Fagerlin, A., Butler, J., Stevens, V., Drews, F. A., Shoemaker, H., ... & Scherer, L. D. (2022). Communicating about COVID-19 vaccine development and safety.*PLOS One, 17*(8), e0272426.https://doi.org/10.1371/journal.pone.0272426

*Torres, C., Ogbu-Nwobodo, L., Alsan, M., Stanford, F. C., Banerjee, A., Breza, E., ... & COVID-19 Working Group. (2021). Effect of physician-delivered COVID-19 public health messages and messages acknowledging racial inequity on Black and White adults’ knowledge, beliefs, and practices related to COVID-19: a randomized clinical trial. *JAMA Network Open, 4*(7), e2117115-e2117115.https://doi.org/10.1001/jamanetworkopen.2021.17115

*van Baal, S. T., Walasek, L., Karanfilovska, D., Cheng, A. C., & Hohwy, J. (2022). Risk perception, illusory superiority and personal responsibility during COVID‐19: An experimental study of attitudes to staying home. *British Journal of Psychology, 113*(3), 608-629.https://doi.org/10.1111/bjop.12554

*Wilke, J., Mohr, L., Yuki, G., Bhundoo, A. K., Jiménez-Pavón, D., Laiño, F., ... & Hespanhol, L. (2022). Train at home, but not alone: a randomised controlled multicentre trial assessing the effects of live-streamed tele-exercise during COVID-19-related lockdowns. *British Journal of Sports Medicine, 56*(12), 667-675.https://doi.org/10.1136/bjsports-2021-104994

*Wilson, D., Driller, M., Johnston, B., & Gill, N. (2021). The effectiveness of a 17-week lifestyle intervention on health behaviors among airline pilots during COVID-19. *Journal of Sport and Health Science, 10*(3), 333-340.https://doi.org/10.1016/j.jshs.2020.11.007

*Wilson, D., Driller, M., Winwood, P., Johnston, B., & Gill, N. (2021). The effects of a brief lifestyle intervention on the health of overweight airline pilots during COVID-19: A 12-month follow-up study. *Nutrients, 13*(12), 4288.https://doi.org/10.3390/nu13124288

*Xiao, Y., & Yu, S. (2022). Using Humor to Promote Social Distancing on Tiktok During the COVID-19 Pandemic. *Frontiers in*

*Psychology, 13*, 887744..https://doi.org/10.3389/fpsyg.2022.887744

*Zhao, Y., Wang, W., Wang, M., Gao, F., Hu, C., Cui, B., ... & Ren, H. (2022). Personalized individual-based exercise prescriptions are effective in treating depressive symptoms of college students during the COVID-19: A randomized controlled trial in China. *Frontiers in Psychiatry, 13*, 1015725.https://doi.org/10.3389/fpsyt.2022.1015725
