# Supplementary material for: Intervention Effectiveness of Health Behaviors During COVID‐19: A Systematic Review and a Network Meta‐Analysis
Source: Psych J. 2025 Sep 29;14(6):841–52. doi: 10.1002/pchj.70054 (PMC12702596; doi:10.1002/pchj.70054)
Supplement: Supplementary file 5 — Data S5: Supporting Information. [file PCHJ-14-841-s003.docx]

S5 Table 1 Results of subgroup analysis

| Moderator | Subgroup | Treat | SMD | *LLCI* | *ULCI* | Z | P |
| --- | --- | --- | --- | --- | --- | --- | --- |
| Country/region | ESP | EI | 0.175 | -1.045 | 1.395 | 0.28 | 0.779 |
|  |  | PI | 0.300 | -0.412 | 1.013 | 0.83 | 0.409 |
|  | KOR | EI | 0.828 | 0.479 | 1.177 | 4.64 | **0.000** |
|  |  | HE | 0.276 | -0.400 | 0.953 | 0.80 | 0.423 |
|  |  | MI | 0.570 | -0.008 | 1.147 | 1.93 | 0.053 |
|  | CHN | EI | 1.863 | 0.540 | 3.186 | 2.76 | **0.006** |
|  |  | PI | 0.000 | -2.277 | 2.277 | 0.00 | 1.000 |
|  | GBR | EI | -0.034 | -0.390 | 0.322 | -0.19 | 0.851 |
|  |  | IMI | 0.015 | -0.054 | 0.084 | 0.42 | 0.675 |
|  |  | MI | 0.187 | -0.004 | 0.379 | 1.92 | 0.055 |
|  | USA | EI | 1.444 | 1.097 | 1.792 | 8.150 | **0.000** |
|  |  | IMI | 0.064 | 0.018 | 0.109 | 2.740 | **0.006** |
|  |  | MI | 0.031 | -0.110 | 0.173 | 0.430 | 0.665 |
|  |  | PI | 0.167 | 0.055 | 0.278 | 2.940 | **0.003** |
|  | KWT | HE | 0.587 | 0.370 | 0.804 | 5.30 | **0.000** |
|  | TWN | HE | 1.286 | 0.856 | 1.716 | 5.86 | **0.000** |
|  |  | MI | 0.001 | -0.744 | 0.746 | 0 | 0.998 |
|  | JPN | IMI | 0.220 | 0.176 | 0.263 | 9.92 | **0.000** |
|  | ES | MI | 0.628 | -0.016 | 1.271 | 1.91 | 0.056 |
|  | NZL | MI | 1.341 | 0.766 | 1.917 | 4.57 | **0.000** |
| Study type | RCT | EI | 0.460 | 0.240 | 0.681 | 4.100 | **0.000** |
|  |  | HE | 0.546 | 0.316 | 0.777 | 4.650 | **0.000** |
|  |  | IMI | 0.073 | -0.535 | 0.682 | 0.240 | 0.813 |
|  |  | MI | 0.520 | 0.268 | 0.772 | 4.050 | **0.000** |
|  |  | PI | 0.209 | -0.160 | 0.577 | 1.110 | 0.267 |
|  | Quasi-  experiment | EI | 4.570 | 4.019 | 5.122 | 16.250 | **0.000** |
|  |  | HE | 0.276 | -0.468 | 1.021 | 0.730 | 0.467 |
|  |  | IMI | 0.087 | 0.042 | 0.131 | 3.810 | **0.000** |
|  |  | MI | 0.583 | 0.423 | 0.742 | 7.150 | **0.000** |
|  |  | PI | 0.165 | -0.020 | 0.351 | 1.750 | 0.080 |
| Outcome | Health behavior | HE | 0.708 | 0.429 | 0.987 | 4.970 | **0.000** |
|  |  | IMI | 0.055 | -0.086 | 0.196 | 0.770 | 0.443 |
|  | Healthy nutrition | HE | 0.305 | -0.229 | 0.839 | 1.120 | 0.262 |
|  |  | MI | 1.653 | 1.140 | 2.165 | 6.320 | **0.000** |
|  |  | PI | 0.666 | -0.300 | 1.633 | 1.350 | 0.176 |
|  | COVID-19 vaccine | HE | 0.761 | 0.502 | 1.021 | 5.750 | **0.000** |
|  |  | IMI | 0.002 | -0.054 | 0.057 | 0.060 | 0.953 |
|  | Prevention intention of COVID-19 | HE | 0.776 | 0.585 | 0.966 | 7.990 | **0.000** |
|  |  | IMI | 0.195 | 0.116 | 0.275 | 4.830 | **0.000** |
|  |  | PI | 0.146 | -0.254 | 0.545 | 0.710 | 0.476 |
|  | Hand washing | HE | -0.817 | -1.267 | -0.367 | -3.560 | **0.000** |
|  |  | IMI | 0.100 | -0.013 | 0.214 | 1.740 | 0.082 |
|  | Physical activity | EI | 0.800 | 0.541 | 1.059 | 6.040 | **0.000** |
|  |  | MI | 0.523 | 0.260 | 0.787 | 3.890 | **0.000** |
|  | Social distancing | IMI | 0.066 | 0.001 | 0.132 | 1.970 | **0.048** |
|  |  | PI | 0.267 | 0.081 | 0.452 | 2.820 | **0.005** |
|  | Mask wearing | IMI | -0.028 | -0.068 | 0.011 | -1.400 | 0.162 |
| Form | Offline | EI | 0.121 | -0.379 | 0.620 | 0.470 | 0.636 |
|  |  | HE | 0.495 | 0.231 | 0.759 | 3.670 | **0.000** |
|  |  | IMI | 0.155 | -0.401 | 0.711 | 0.550 | 0.584 |
|  |  | MI | 0.990 | 0.706 | 1.273 | 6.850 | **0.000** |
|  | Online | EI | 0.730 | 0.581 | 0.879 | 9.620 | **0.000** |
|  |  | HE | 0.554 | 0.338 | 0.769 | 5.030 | **0.000** |
|  |  | IMI | 0.087 | 0.031 | 0.143 | 3.050 | **0.002** |
|  |  | MI | 0.288 | 0.118 | 0.458 | 3.330 | **0.001** |
|  |  | PI | 0.185 | 0.033 | 0.337 | 2.380 | **0.017** |
| behavior | Health-promoting behavior | EI | 0.754 | 0.554 | 0.953 | 7.400 | **0.000** |
|  |  | HE | 0.511 | 0.234 | 0.789 | 3.610 | **0.000** |
|  |  | IMI | -0.003 | -0.268 | 0.262 | -0.020 | 0.983 |
|  |  | MI | 0.643 | 0.458 | 0.829 | 6.790 | **0.000** |
|  |  | PI | 0.305 | -0.206 | 0.816 | 1.170 | 0.242 |
|  | Preventive health behavior | HE | 0.517 | 0.338 | 0.696 | 5.660 | **0.000** |
|  |  | IMI | 0.099 | 0.052 | 0.145 | 4.160 | **0.000** |
|  |  | PI | 0.147 | 0.006 | 0.289 | 2.040 | **0.041** |

S5 Table 2 Results of meta-regression analysis

| Covariate | Coefficient | SE | Z | P | *LLCI* | *ULCI* |
| --- | --- | --- | --- | --- | --- | --- |
| Exercise intervention | | | | | | |
| Age | -0.017 | 0.0069 | -2.4595 | **0.0139** | -0.0306 | -0.0035 |
| Gender | 0.0013 | 0.0131 | 0.0973 | 0.9225 | -0.0245 | 0.027 |
| Quality | 7.0841 | 1.3317 | 5.3197 | **0.0001** | 4.4741 | 9.6941 |
| Health education | | | | | | |
| Age | -0.0129 | 0.0057 | -2.2687 | **0.0233** | -0.024 | -0.0018 |
| Gender | -0.0004 | 0.0033 | -0.1248 | 0.9007 | -0.0068 | 0.006 |
| Quality | 0.2363 | 1.3578 | 0.174 | 0.8619 | -2.4251 | 2.8976 |
| Mixed intervention | | | | | | |
| Age | -0.0036 | 0.0088 | -0.4097 | 0.6821 | -0.0208 | 0.0136 |
| Gender | -0.0081 | 0.0069 | -1.1889 | 0.2345 | -0.0216 | 0.0053 |
| Quality | -0.3744 | 1.0824 | -0.3459 | 0.7294 | -2.4958 | 1.7471 |
| Psychological intervention | | | | | | |
| Age | 0.0121 | 0.0096 | 1.254 | 0.2099 | -0.0068 | 0.0309 |
| Gender | -0.0121 | 0.0077 | -1.5827 | 0.1135 | -0.0271 | 0.0029 |
| Quality | 0.2493 | 0.4605 | 0.5413 | 0.5883 | -0.6533 | 1.1519 |
| Information intervention | | | | | | |
| Age | -0.0032 | 0.0036 | -0.884 | 0.3767 | -0.0102 | 0.0039 |
| Gender | 0.0008 | 0.0017 | 0.4938 | 0.6215 | -0.0025 | 0.0042 |
| Quality | -0.475 | 0.1238 | -3.8368 | **0.0001** | -0.7177 | -0.2324 |
